# Bipolar disorders distribution in the two genders: An analysis of recently published large sample studies

**DOI:** 10.1192/j.eurpsy.2021.524

**Published:** 2021-08-13

**Authors:** R. Cafaro, B. Dell’Osso, T. Ketter

**Affiliations:** 1 Department Of Biomedical And Clinical Sciences Luigi Sacco, University of Milan, Milan, Italy; 2 Department Of Psychiatry And Behavioural Sciences, Stanford University, Stanford, United States of America

**Keywords:** Bipolar Disorders, Gender, prevalence

## Abstract

**Introduction:**

In the last decade, literature reports evidences of a growing number of patients diagnosed with Bipolar Disorders (BD), however, only few data are available regarding the distribution of BD diagnosis in the two genders. In fact, although many studies show differences in presentation and comorbidities of BD in the two genders, BD are commonly perceived as equally affecting both women and men. On the other hand, BD in female patients can often be misdiagnosed as MDD, especially because of the higher number of depressive episodes that characterize BD in women.

**Objectives:**

We aimed to analyse the gender composition of large samples, recently published studies on BD, in order to evaluate a possible modification of representation of BD in the two genders.

**Methods:**

An electronic review of literature was conducted, and results were filtered by year of publication (2011-2020) and number of patients (> 1,000).

**Results:**

Our results show a higher number of female patients in every study evaluated (N=10). Of note, we found a higher number of females also in BD-I subsamples, in contradiction with previously published literature.

**Conclusions:**

Even if with limitations connected to the design of the study, our study supports the hypothesis of a gender specific increment in BD diagnosis, and could lead the way for large epidemiological studies assessing gender specific prevalence of BD in the general population. Given the risks connected with untreated BD, and with antidepressants monotherapy, a better understanding of BD epidemiology could help physicians adequately diagnose and treat affected subjects.

